# Adequacy of antibiotic prophylaxis and incidence of surgical site infections in neck surgery

**DOI:** 10.1038/s41598-021-95831-9

**Published:** 2021-08-12

**Authors:** M. Alonso-García, A. Toledano-Muñoz, J. M. Aparicio-Fernández, F. M. De-la-Rosa-Astacio, D. Rodríguez-Villar, A. Gil-de-Miguel, M. Durán-Poveda, G. Rodríguez-Caravaca

**Affiliations:** 1 Department of Preventive Medicine, Alcorcón Foundation University Hospital, C/ Budapest 1, 28922 Alcorcón, Madrid Spain; 2 Department of Medical Specialities and Public Health, Rey Juan Carlos University, Alcorcón, Madrid Spain; 3 Department of Otorhinolaryngology, Alcorcón Foundation University Hospital, Alcorcón, Madrid Spain

**Keywords:** Microbiology, Health care, Medical research, Risk factors

## Abstract

Health care-related infections are frequent and among them surgical site infection (SSI) are the most frequent in hospitals. The objective was to evaluate the adequacy of antibiotic prophylaxis in patients undergoing neck surgery and its relationship with the incidence of surgical site infection (SSI). Prospective cohort study. The adequacy of antibiotic prophylaxis in patients undergoing neck surgery was evaluated. Antibiotic prophylaxis was considered adequate when it conformed to all items of the protocol (antibiotic used, time of administration, administration route, dose and duration). The cumulative incidence of SSI was calculated, and the relationship between SSI and antibiotic prophylaxis adequacy was determined using adjusted relative risk (RR). Antibiotic prophylaxis was administered in 63 patients and was adequate in 85.7% (95% CI 75.0–92.3) of them. The cumulative incidence of SSI was 6.4% (95% CI 3.4–11.8). There was no significant relationship between antibiotic prophylaxis inadequacy and the incidence of SSI (RR = 2.4, 95% CI 0.6–10.6). Adequacy of antibiotic prophylaxis was high and it did not affect the incidence of SSIs.

## Introduction

Surgical site infection (SSI) is the most common healthcare-associated infection (HAI)^1,2^. The incidence of SSI depends on variables that are both intrinsic (sex, age, comorbidities, etc.) and extrinsic (duration and type of surgery, surgeon experience, preoperative preparation of the patient, etc.) to the patient^3,4^ and ranges from 1 and 20% depending on the type of surgical procedure^5^.

Neck surgery is one of the most important types of otorhinolaryngological surgery. Specific studies that estimate the incidence of SSI in neck surgery report incidences of less than 1% when the surgery is clean^6,7^ and between 25 and 85% when the surgery is clean-contaminated^8,9^. These types of infections are of great health importance because they increase the average length of hospital stay, morbidity and mortality, and the healthcare cost per patient^10,11^.

One of the most effective measures for reducing SSI is antibiotic prophylaxis, which is indicated in clean-contaminated and contaminated surgeries and in clean surgeries in which an implant is placed, the operative time is prolonged, the patient is immunosuppressed or that are performed on a specific site^12,13^. The purpose of preoperative prophylaxis is for the antibiotic to reach an optimal concentration in the tissue during the surgical procedure and in the hours immediately following the closure of the incision. The choice of antibiotic will depend on the typical flora of each healthcare centre, the resistance map and the surgical site. For long surgeries, when the duration exceeds twice the half-life of the antibiotic, the dose should be repeated^14,15^.

The use of antibiotic prophylaxis protocols facilitates adequacy, and in our hospital, we have a protocol that is periodically updated based on the recommendations in the literature. The protocol is reviewed and updated every two years by the infections commission.

The objective of our study was to evaluate the adequacy of the antibiotic prophylaxis to our protocol in patients undergoing neck surgery and the relationship between the adequacy of antibiotic prophylaxis and the incidence of SSI.

## Methods

A prospective cohort study was conducted from January 2011 to December 2019. Consecutive patients over 18 years old who underwent neck surgery at the Alcorcón Foundation University Hospital (Hospital Universitario Fundación Alcorcón—HUFA) were sampled. Patients who at the time of surgery had an active infection or were receiving antibiotic treatment were excluded. The study was approved by the Ethics and Clinical Research Committee of HUFA (number 9/14).

The sample size was calculated for an estimated proportion of compliance with the antibiotic prophylaxis protocol of 85%, a confidence level of 95%, an accuracy of 10% and considering losses of 5%. Thus, it was considered necessary to include 52 patients.

Sociodemographic variables (age, sex), patient comorbidities (cancer, diabetes, chronic obstructive pulmonary disease (COPD), obesity, liver cirrhosis, malnutrition, addiction to parenteral drugs, neutropenia, kidney failure, immunodeficiency), surgical risk according to the American Society of Anaesthesiologists (ASA) classification, surgery-related variables (type of surgery, duration, International Classification of Diseases (ICD-9-CM) diagnosis, drainage, shaving or transfusion), antibiotic prophylaxis-related items (election, time of administration, administration route, dose, duration), compliance with the protocol and SSI-related variables (diagnosis of infection, microorganism and location) were considered. Antibiotic prophylaxis stated in the hospital protocol is shown in Table 1 and it was prescribed for all otorhinolaryngological surgery undergoing laryngectomy or oral or pharyngeal mucosal incision, either in clean, clean-contaminated or contaminated surgery. Antibiotic prophylaxis was considered adequate when it conformed to all items of the protocol. In case of protocol inadequacy, the reason for inadequacy was recorded as follows: time of administration (the dose was not administered 30–60 min before surgery), election (the antibiotic administered differed from that specified in the protocol), duration (prophylaxis was prescribed more than 24 h), administration route (administered by a route other than intravenous) or dose (the dose was different from that described in the protocol). Compliance was defined as the percentage of antibiotic prophylaxis administered when indicated. The diagnosis of SSI was made based on the Centres for Disease Control and Prevention (CDC) criteria^16^ and was evaluated jointly by the Preventive Medicine Unit and the Otorhinolaryngology Service. The surgical procedures included are shown in Table 2 and ICD-9-CM codes 30.22, ‘Vocal cordectomy’, and 31.45, ‘Open biopsy of the larynx and trachea’, were classified as clean surgeries.

**Table 1:** Antibiotic prophylaxis protocol in otorhinolaryngological surgery.

	Antibiotic	Time
Standard	Cefazolin 2 g iv or amoxicillin-clavulanic acid 2 g iv	Prior to anaesthetic induction
In cases of allergy to beta-lactams	Clindamycin 600 mg iv + gentamicin 3–5 mg/kg/24 h	Prior to anaesthetic induction

**Table 2:** Distribution of surgical interventions.

ICD-9-CM code	Intervention	N	%
30.1	Hemilaryngectomy	1	0.7
30.21	Epiglottectomy	0	0
30.22	Vocal cordectomy	56	40
30.29	Other partial laryngectomy	10	7.1
30.3	Total laryngectomy	21	15
30.4	Radical laryngectomy	8	5.7
31.45	Open biopsy of larynx and trachea	8	5.7
40.40	Radical neck dissection	4	2.9
40.41	Radical neck dissection, unilateral	29	20.7
40.42	Radical neck dissection, bilateral	3	2.2
Total		140	100.0

Clinical follow-up of patients was performed from the time of surgery to 30 days after, which is the maximum incubation period for SSIs in surgeries not involving implant placement. They were actively monitored from the time of surgery until discharge and data were collected using an ad hoc form from the following data sources: hospital electronic medical records for surgery and hospital stay-related variables, in-hospital readmission, outpatients’ revision and care provided in the hospital’s emergency department. HORUS, the clinical history platform of the Community of Madrid for primary health-care was also used, which allowed follow-up through primary care visits from discharge to the end of the incubation period.

### Statistical analysis

A descriptive analysis of the quantitative variables was performed using the mean and standard deviation or the median and interquartile range (IQR) if the variables were not normally distributed; these variables were compared using Student’s t test or the Mann–Whitney test in cases of nonnormality. Qualitative variables were described using frequency distributions and percentages and were compared using the Pearson χ^2^ test or Fisher’s exact test, as appropriate. The cumulative incidence of SSI was calculated, as was the relationship between SSI and antibiotic prophylaxis adequacy using the relative risk (RR). Statistical analysis was performed in SPSS 23.0, and a p-value < 0.05 was considered statistically significant.

### Informed consent

This study was done in accordance with the strengthening the reporting of observational studies in epidemiology (STROBE) statement. Informed consent was obtained from all subjects.

## Results

During the study period, 140 patients were included in the sample and all of the patients completed the 30-day follow up. The mean age was 65.8 ± 12.4 years. A total of 81.4% of the patients were male. Table 2 shows the surgical interventions performed and their distribution frequency. The demographic and clinical characteristics of the patients are summarized in Table 3. The median length of hospital stay was 3 days (IQR 1–14 days), and discharges were almost completely due to cure (97.9%).

**Table 3:** Demographic, clinical and surgical characteristics of patients undergoing head and neck surgery (N = 140).

Variable	N (%)
Mean age (years)/standard deviation	65.7 (12.5)
**Intrinsic and extrinsic factors**	
Cancer	115 (82.7)
Diabetes	41 (29.5)
Chronic obstructive pulmonary disease	26 (18.7)
Obesity	11 (7.9)
Liver cirrhosis	6 (4.3)
Malnutrition	6 (4.3)
Renal insufficiency	4 (2.9)
Immunodeficiency	1 (0.7)
Drainage	54 (38.8)
Shaving	7 (5.0)
Transfusion	1 (0.7)
**Surgery characteristics**	
American Society of Anesthesiologists index	
ASA 1	5 (3.6)
ASA 2	74 (53.2)
ASA 3	56 (40.3)
ASA 4	4 (2.9)
Duration of surgery (min)/standard deviation	144 (118)
Wound class	
Clean	64 (45.7)
Clean-contaminated	76 (54.3)

No patient undergoing neck surgery had an addiction to parenteral drugs or neutropenia. Most of the admissions were scheduled (94.3%). The mean duration of surgery was 144 ± 118 min. The surgical checklist was properly completed for 66.5% of patients.

**Figure 1:**
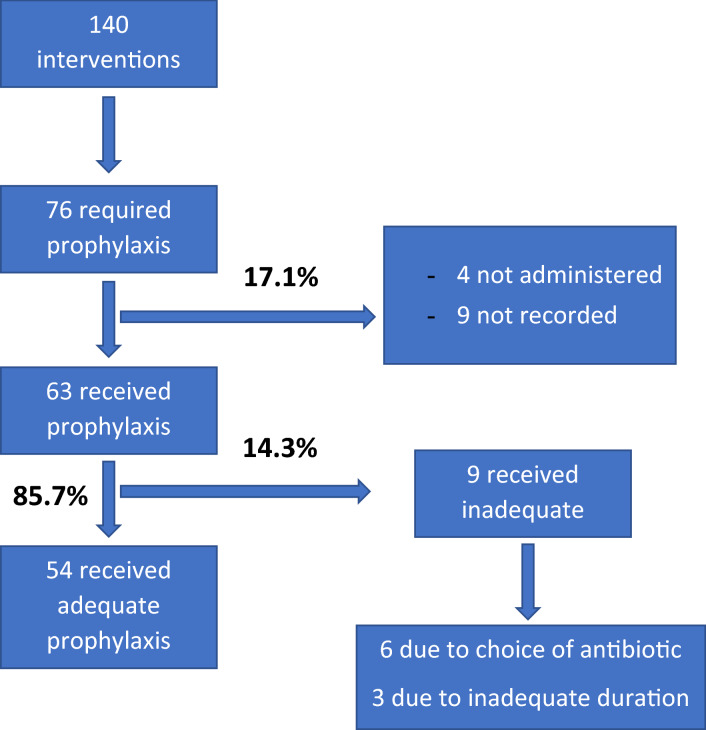
Study outline.

One patient in whom antibiotic prophylaxis was not indicated received cefazolin. The administration of antibiotic prophylaxis was indicated for 76 patients and was administered for 63 (compliance 83%). For 4 patients, prophylaxis was not administered, and for 9 patients, data on this variable were not recorded. The administered prophylaxis was adequate for 85.7% of patients (95% CI 75.0–92.3) (Fig. 1). The most frequent cause of inadequate antibiotic prophylaxis was the choice of antibiotic (9.5%, 95% CI 4.4–19.3), followed by duration (4.8%, 95% CI 1.6–13.1) (Table 4). The most commonly used prophylactic antibiotic was amoxicillin-clavulanate (76.2%), followed by cefazolin (11.1%), clindamycin (6.3%), ciprofloxacin (3.2%), gentamicin (1.6%) and vancomycin (1.6%).

**Table 4:** Compliance and adequacy of antibiotic prophylaxis use to the hospital protocol (n = 76, compliance 83%).

Adequacy of antibiotic prophylaxis	n	%	95% CI
Adequate	54	85.7	75.0–92.3
Inadequate (duration)	3	4.8	1.6–13.1
Inadequate (election)	6	9.5	4.4–19.3
Inadequate (route)	0	0	–
Inadequate (dose)	0	0	–
Inadequate (time of administration)	0	0	–
Total	63	100%	–

Nine patients were diagnosed with SSI, with an overall cumulative incidence of 6.4% (95% CI 3.4–11.8). One of the infections occurred in the group of patients who did not receive prophylaxis The incidence of SSI in the group of patients with adequate antibiotic prophylaxis was 9.2% (95% CI 1.5–17.0), lower than that of 22.2% (95% CI 0.0–49.4) in the group of patients with inadequate antibiotic prophylaxis. Two of the patients who did not received prophylaxis had a SSI, with an overall cumulative incidence of 2.5% (95% CI 0.0–6.0).

**Figure 2:**
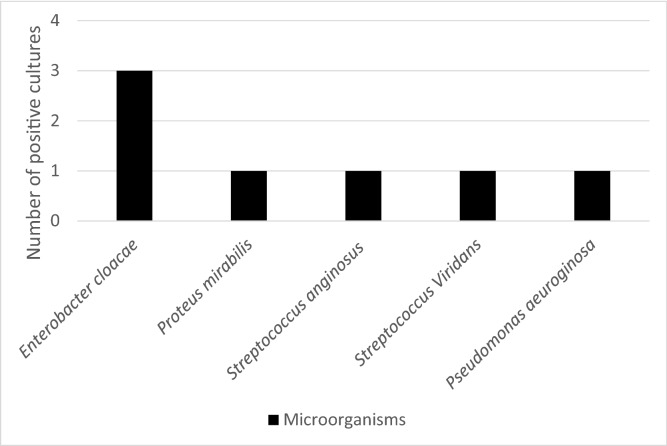
Surgical Site Infection aetiology.

In terms of location, six SSIs were superficial, and three were deep. The most frequently isolated microorganism was *Enterobacter cloacae* (Fig. 2).

SSI occurred with a mean of 14.0 ± 16.1 days since the operation date. SSI was diagnosed in-hospital in seven patients and in the first week after discharge in two patients. Patients without SSI had a median length of hospital stay of 3 days (IQR 1–13 days), while for patients with SSI, the median stay was 26 days (IQR 15.5–41 days) (p < 0.05).

There was no significant relationship neither between inadequate antibiotic prophylaxis and the incidence of SSI (RR = 2.4, 95% CI 0.6–10.6) nor between patients with or without indication of prophylaxis and the incidence of SSI (RR = 0.23, 95% CI 0.05–1.1).

## Discussion

A low incidence of SSI is directly related to an adequate surgical technique and is commonly used as an indicator of quality and safety in hospital care^17^. The incidence of SSI in this study was 6.4%, higher than the incidence reported by the CDC (3.5%)^18^ and lower than that reported in other studies^8,9,19–21^, which have described an incidence of up to 85%. Our patients were mostly men (84% men and 16% women) with a mean age of 65.8 years, in line with what has been reported in other neck surgery studies^22^.

Up to 60% of SSIs can be prevented with evidence-based clinical practices such as preoperative preparation or optimal antibiotic selection^12,23,24^. In neck surgery, there are few studies that evaluate the effect of antibiotic prophylaxis on the incidence of SSI. The results of our study showed a non-significant reduction in SSIs as a function of the administration of adequate antibiotic prophylaxis. There is some controversy between studies that show this relationship^25,26^ and others that do not show the efficacy of antibiotic prophylaxis^27^, although a recent systematic review by Vander Porten et al.^28^ concludes that the administration of antibiotic prophylaxis clearly reduces the incidence of SSIs in clean-contaminated surgery.

The administration of the prophylactic antibiotic between 30 and 60 min before the surgical incision reduces SSIs and should be repeated if the surgery lasts more than 4 h^29^. In our study, antibiotic prophylaxis was indicated for 67 patients (47.9%), and its administration was adequate for 85.7% (95% CI 75.0–92.3) of them. The surgeries for which antibiotic prophylaxis was not indicated were those classified under ICD-9-CM codes 30.22, “Vocal cordectomy”, and 31.45, “Open biopsy of the larynx and trachea”, which are classified as clean surgeries and for which the efficacy of antibiotic prophylaxis has not been observed^30,31^. Two patients in our series who developed SSI received inadequate prophylaxis, and the percentage of patients who did not receive prophylaxis, even when indicated, was 6.0%, which was lower than in other studies^32,33^ and higher than that reported by Hemssen et al.^34^. Our percentage of adequacy (85.7%) was high; however, it was lower than that reported in other studies^35^, higher than that of a study conducted in our area^36^ and in line with that of Takahashi et al.^37^. The main cause of inadequacy of the antibiotic prophylaxis protocol in our study was the choice of antibiotic, which is the most frequently reported cause of inadequacy^38^.

The microorganisms isolated from SSIs during neck surgery are very diverse; the most frequent are *Staphylococcus aureus*, *Klebsiella pneumoniae*, *Pseudomonas aeruginosa,* enterobacteria and *Enterococcus* spp.^23,39,40^, which are common colonizing microorganisms of the airways. In our study, most of the microorganisms were gram negative (*Enterobacter cloacae*), which is consistent with other studies that describe this group of microorganisms as the most frequently identified in postoperative infections in neck surgery^41^.

Monitoring the adequacy of the items of the antibiotic prophylaxis protocols can help controlling SSIs and the antibiotics to be effective, as shows different previous research^35,38,42^.

The possible limitations of the study include the small sample size, although a sample size estimation was performed to ensure that the percentage of antibiotic prophylaxis adequacy was adequately estimated. The incidence of infection may be underestimated by the loss of exceptional cases of superficial infections with minimal symptoms for which the patient did not contact the emergency department, the otorhinolaryngology service or the primary care doctor. We believe that this bias has been controlled because the patients were followed during admission and after discharge and their attendance at different care facilities was prospectively evaluated for 30 days, the maximum incubation period of an SSI in surgeries without implant placement.

We can conclude that antibiotic prophylaxis is one of the most cost-effective measures for reducing the incidence of SSIs, and compliance with existing protocols is a measure that is used to assess the quality of health care. Adequacy of antibiotic prophylaxis in our study was high, and the main cause of inadequacy was the choice of antibiotic. We observed a reduction in the incidence of SSI during neck surgery in patients who were adequately administered prophylaxis, although this relationship was not statistically significant. So, we would recommend to monitor the adequacy of the items of the antibiotic prophylaxis protocols, in order to control SSIs.

## Data Availability

The datasets generated and analyzed during the current study are not publicly available. Because they are archived in the clinical databases of the Alcorcón Foundation University Hospital they are only used for scientific purposes. Datasets are available from the corresponding author upon justified request.
